# Potential of Marine Terpenoids against SARS-CoV-2: An *In Silico* Drug Development Approach

**DOI:** 10.3390/biomedicines9111505

**Published:** 2021-10-20

**Authors:** Alaka Sahoo, Shivkanya Fuloria, Shasank S. Swain, Sujogya K. Panda, Mahendran Sekar, Vetriselvan Subramaniyan, Maitreyee Panda, Ajaya K. Jena, Kathiresan V. Sathasivam, Neeraj Kumar Fuloria

**Affiliations:** 1 Department of Skin & VD, Institute of Medical Sciences and SUM Hospital, Siksha ‘O’ Anusandhan Deemed to be University, Bhubaneswar 751003, Odisha, India; alakasahoo81@gmail.com (A.S.); pandamaitreyee@gmail.com (M.P.); ajayajenabbsr@gmail.com (A.K.J.); 2 Faculty of Pharmacy, Centre of Excellence for Biomaterials Engineering, AIMST University, Bedong 08100, Kedah, Malaysia; 3 Division of Microbiology and NCDs, ICMR–Regional Medical Research Centre, Bhubaneswar 751023, Odisha, India; swain.shasanksekhar86@gmail.com; 4 Center of Environment Climate Change and Public Health, Utkal University, Vani Vihar, Bhubaneswar 751004, Odisha, India; sujogyapanda@gmail.com; 5 Department of Pharmaceutical Chemistry, Faculty of Pharmacy and Health Sciences, Universiti Kuala Lumpur Royal College of Medicine Perak, Ipoh 30450, Perak, Malaysia; mahendransekar@unikl.edu.my; 6 Faculty of Medicine, Bioscience and Nursing, MAHSA University, Jalan SP 2, Bandar Saujana Putra, Jenjarom 42610, Selangor, Malaysia; drvetriselvan@mahsa.edu.my; 7 Faculty of Applied Science, Centre of Excellence for Biomaterials Engineering, AIMST University, Bedong 08100, Kedah, Malaysia; skathir@aimst.edu.my

**Keywords:** marine terpenoids, SARS-CoV-2, molecular docking, toxicity and drug−likeness profiles

## Abstract

In an emergency, drug repurposing is the best alternative option against newly emerged severe acute respiratory syndrome coronavirus-2 (SARS-CoV-2) infection. However, several bioactive natural products have shown potential against SARS-CoV-2 in recent studies. The present study selected sixty-eight broad-spectrum antiviral marine terpenoids and performed molecular docking against two novel SARS-CoV-2 enzymes (main protease or M^pro^ or 3CL^pro^) and RNA-dependent RNA polymerase (RdRp). In addition, the present study analysed the physiochemical-toxicity-pharmacokinetic profile, structural activity relationship, and phylogenetic tree with various computational tools to select the ‘lead’ candidate. The genomic diversity study with multiple sequence analyses and phylogenetic tree confirmed that the newly emerged SARS-CoV-2 strain was up to 96% structurally similar to existing CoV-strains. Furthermore, the anti-SARS-CoV-2 potency based on a protein−ligand docking score (kcal/mol) exposed that the marine terpenoid brevione F (−8.4) and stachyflin (−8.4) exhibited similar activity with the reference antiviral drugs lopinavir (−8.4) and darunavir (−7.5) against the target SARS−CoV−M^pro^. Similarly, marine terpenoids such as xiamycin (−9.3), thyrsiferol (−9.2), liouvilloside B (−8.9), liouvilloside A (−8.8), and stachyflin (−8.7) exhibited comparatively higher docking scores than the referral drug remdesivir (−7.4), and favipiravir (−5.7) against the target SARS-CoV-2−RdRp. The above *in silico* investigations concluded that stachyflin is the most ‘lead’ candidate with the most potential against SARS-CoV-2. Previously, stachyflin also exhibited potential activity against HSV-1 and CoV-A59 within IC_50_, 0.16–0.82 µM. Therefore, some additional pharmacological studies are needed to develop ‘stachyflin’ as a drug against SARS-CoV-2.

## 1. Introduction

Coronavirus disease−2019 (COVID-19) has been considered one of the most destructive pandemics in the 21st century [1]. The uncertain outbreak and rapid transformation with a higher morbidity and mortality rate create a global health emergency [2]. The WHO reports >233 million infected cases and >4.7 million deaths from SARS-CoV-2 up to 1st October 2021 [1]. Recent reports suggest that among the coronavirus family, the mutated SARS-CoV-2 strain single−stranded RNA virus (ssRNA) spreads much faster when compared with previously reported SARS and Middle East Respiratory Syndrome (MERS) [3,4]. Evidence suggests that approximately 75–80% of genomic similarity between current strains and existing CoV strains is crucial for rapid drug and vaccine development [3,4,5]. Facts suggest that the UK−based SARS-CoV-2 (B.1.1.7), South Africa−based (B.1.351 and C.1.2), and India−based (B.1.617.2) are the most contagious variants. The B.1.617.2 (delta) is considered a super−spreader infectious strain with higher mortality rates in India and several other Asian continents [1]. Identifying or locating potential−cum−putative therapeutic agents is a challenging task [6,7,8,9]. In contrast, computational−based or artificial−intelligence drug discovery platform plays a crucial role in finding several ‘lead drug candidates’ in minimum time and cost to accelerate the drug development and further pharmacological validation.

From a drug discovery point of view, main protease (M^pro^), spike glycoprotein (S−protein), RdRp, envelop protein (E−protein), membrane protein (M−protein), and nucleocapsid (N−protein) are some important drug targets that are associated with viral−host interaction for assembly, and formation/replication of virus genomes in the host cell [10,11,12,13]. In addition, there are several ideal targets in the host side, namely, angiotensin−converting enzyme 2 (ACE2) and receptor transmembrane serine protease 2 (TMPRSS2), which plays a crucial role during viral entry and fusion of the viral genome [4,13]. Overall, M^pro^ and RdRp are the two putative targets for drug development [13,14].

The FDA’s drug approval rate has decreased in the last two decades because most synthetic drug entities/candidates commonly fail in clinical for side effects or higher toxicity profiles [15,16,17,18]. Indeed, from the history of drug discovery, natural products always play a significant role as a parental source for mainstream medicine attributed to their multi−factorial activity and lesser toxicity. Marine diversity is a substantial resource for potential bioactive chemical constituents with a wide range of therapeutic applications. Antibacterial (e.g., aztreonam, tetracycline, erythromycin, imipenem, vancomycin, etc.), antiviral (e.g., vidarabine), anticancer (e.g., brentuximab vedotin, ecteinascidin 743, etc.), and antitubercular agents (e.g., rifampicin) are some notable mainstream drugs derived from marine chemosphere [19,20,21,22].

The present study selected sixty-eight antiviral terpenoids isolated from marine algae, soft coral, sponge, sea cucumber, fungus, bacteria, etc., from an extensive literature search (Table 1). Then, we have assessed the anti-SARS-CoV-2 potency of each terpenoid through an advanced molecular docking study using two putative drug targets, M^pro^ and RdRp. In addition, various bioinformatics and cheminformatics tools analyzed the potency, drug−ability, and toxicity profiles of terpenoids [23,24,25,26]. Previously, selected terpenoids exhibited broad-spectrum potent antiviral activity (IC_50_/EC_50_) against coronavirus−19 (CoV-19), antienterovirus 71 (EV71), influenza subtype H1N1 or swine flu (H1N1), human cytomegalovirus (HCMV), human immune deficiency virus (HIV-1), human metapneumovirus (HMPV), herpes simplex-1 virus (HSV-1), rhinovirus, and vesicular stomatitis virus (VSV), etc. from different enzymatic assays (Table 1).

## 2. Material and Methods

### 2.1. Ligand and Receptor Preparation

**Figure 1:**
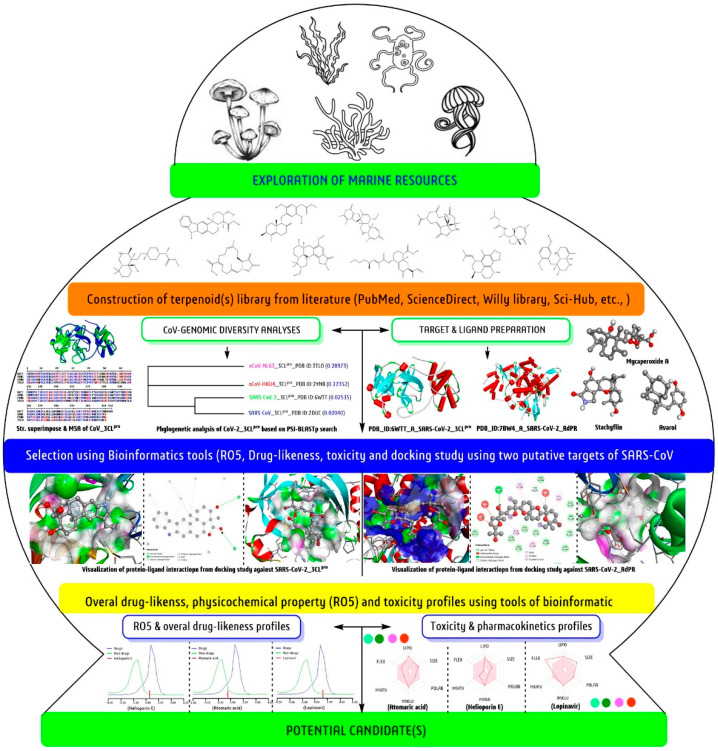
A schematic presentation for screening of most potential anti-SARS-CoV-2 candidates from selected sixty–eight marine terpenoids using bioinformatics tool.

In the current study, a total of sixty−eight antiviral marine terpenoids with active concentration (IC_50_/EC_50_ in µM) against the specific virus were selected from previously published literature (Table 1). Each terpenoid as a therapeutical candidate is known as a ligand during the computational investigation against SARS-CoV-2. The chemical structures were retrieved with individual database IDs and simplified molecular−input line−entry system (SMILES) code. Other relevant information on ligands was collected from the PubChem (https://pubchem.ncbi.nlm.nih.gov; assessed on 25 December 2020) and ChemSpider database (http://www.chemspider.com/ assessed on 25 December 2020). Primarily the retrieved terpenoids as ligands in (.sdf) and (.mol) file formats from databases and then converted to one of the widely accepted formats, i.e., (.pdb) by adding explicit hydrogens to it using the software BIOVIA Discovery Studio Visualizer−2019 (BIOVIA-DSV-2019 (Academic version, San Diego, California, USA) [26,66]. Next, the energy minimization and geometry optimization with the Universal force field and Steepest Descent algorithm using Avogadro software to maintain the ligand structure stability and error−free was carried out. Four reference antiviral drugs, darunavir (PubChem ID: 213039) and lopinavir (PubChem ID: 92727) as viral protease inhibitors and favipiravir (PubChem ID: 492405) and remdesivir (PubChem ID: 121304016) as RdRp inhibitors were used (Figure 1).

Furthermore, we have retrieved three−dimensional (3-D) crystallographic protein structures of M^pro^ (PDB ID: 6WTT, composed with 310 amino acids at 2.15 Å resolution) and RdRp (PDB ID: 7BW4, composed with 923 amino acids at 3.70 Å resolution) from the protein data bank (https://www.rcsb.org/; assessed on 25 January 2021) were used as targets for molecular docking study. Then, separate the chain−A structure and removed attached water molecules, ligands before docking study. From literature and structural analysis with BIOVIA-DSV-2019 confirmed that M^pro^ (6WTT) is a homodimer protein structure that contains a ligand with other heteroatoms [67]. Thus, as per the attached ligand site interactions, we have selected nearly the same X-, Y- and Z-coordinates within 54 × 68 × 60 dimensions with a grid spacing of 0.375 Å. The specified coordinates mostly covered the catalytic active site residues, from 40 to 190 position amino acids of 6WTT during docking study. Similarly, the large size RdRp (7BW4) was analyzed and found no ligand attachment in the retrieved structure. As per literature information, the RdRp structure contains seven critical catalytic motifs (A–G), including the core catalytic subunit, nsp12 bound with nsp7−nsp8 heterodimer binding site. Thus, we have mainly targeted the entire residues and designed 116 × 118 × 120 dimension with 0.615 Å spacing grid box covering amino acids residues, 60 (nsp7) to 761 (the critical catalytic residues of motif C) [68]. Then, we docked each ligand in the above-specified grid box and recorded the docking score individually.

In addition, we have also analysed the structural elements, such as percent and position of α-helix, extended strand, β-turn, random coil, etc., taking protein sequence of both targets with protein secondary structure analysis tool, SOPMA (https://npsa-prabi.ibcp.fr/cgi-bin/npsa; assessed on 10 February 2021). As a result, the retrieved SARS-CoV-2-Mpro structure consisted of 24.19% α-helix, 26.77% an extended strand, 10% β-turns, and 39.03% a random coil. Similarly, the SARS-CoV-2-RdRp contained 41.60% α-helix, 19.50% an extended strand, 7.69% β-turn, and 31.20% random coil. After ligand and target structure preparation, a systematic computer-aided drug design (CADD) module was followed to select the potential ‘lead’ against SARS-CoV-2 (Figure 1).

### 2.2. SARS-CoV-2: Genomic−Diversity and Phylogenetic Tree Analyses

Genomic diversity analysis is one of the crucial aspects in drug discovery to know the impact of genetic mutations, target identification, and pathogenesis before developing any lead drug candidates. Therefore, the genomic diversity of newly emerged SARS-CoV-2 was studied to understand the inherited property and genetic differentiation from previously reported CoV strains. Thus, we have projected the genomic similarity (in percentage) of emerging SARS-CoV-2 with previously reported strains of the CoV family through computational tools. Then analysed genomic diversity in the form of sequence and structural similarity (homology protein model) with their distinct taxa with PSI−BLAST (Position−Specific Iterated−BLAST) method using the tool Phyre2 (http://www.sbg.bio.ic.ac.uk/phyre2/; assessed on 10 February 2021) and ClustalOmega (https://www.ebi.ac.uk/tools/msa/clustalo/; assessed on 10 February 2021).

**Figure 2:**
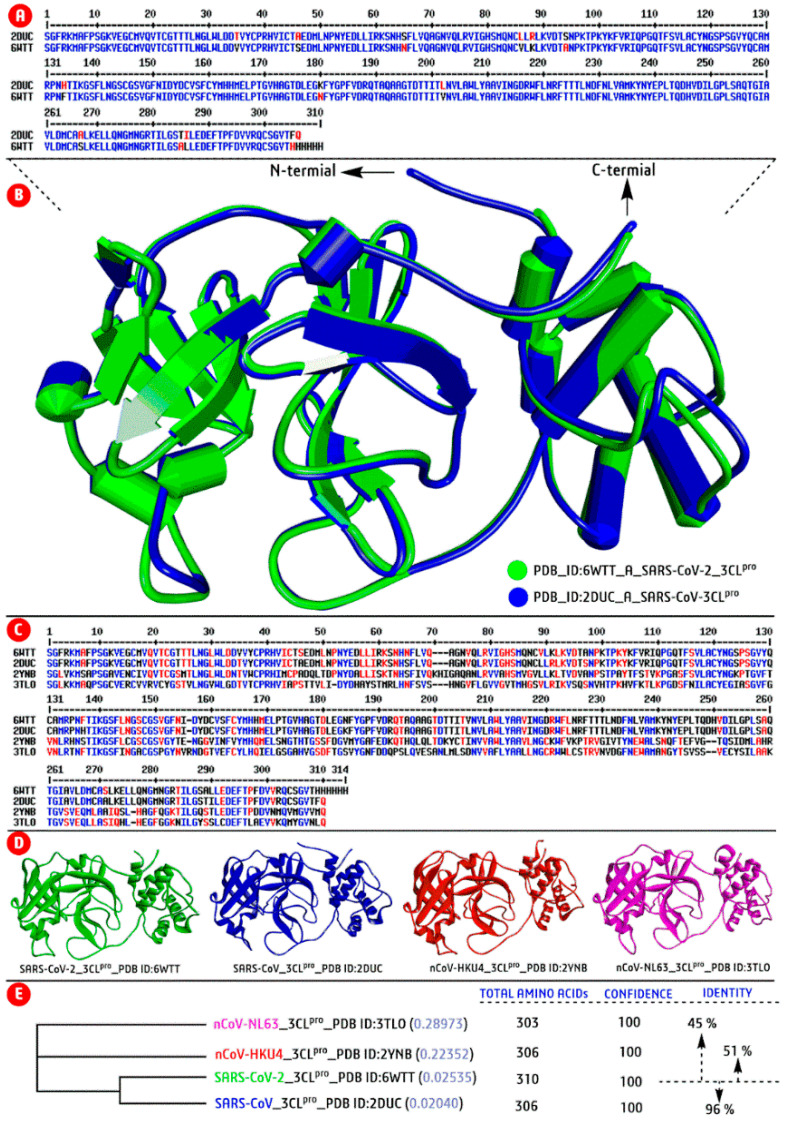
Analysis of genomic diversity SARS-CoV-2 taking sequence and structure of M^pro^ enzyme. (**A**) sequence−level analysis of SARS-CoV (PDB ID: 2DUC) as the most similarity with SARS-CoV-2 (PDB ID: 6WTT) from PSI-blast; (**B**) Structural superimpose of both SARS-CoV-M^pro^ and SARS-CoV-2−M^pro^; (**C**) Multiple sequence analysis between top four homology models during PSI−blast and (**D**) Three−dimensional protein visualization of four similarity structure of M^pro^ in a different color; and (**E**) Phylogenetic analysis with chosen top four homology models by the Neighbour-Joining method to know the genomic similarity and inherited feature of currently emerged SARS-CoV-2.

From extensive sequence−structure analyses, we found that the target SARS-CoV-2−M^pro^ structure (PDB ID: 6WTT) has 96% identical to the previously reported SARS-CoV−M^pro^ structure (PBD ID: 2DUC), 51% equivalent with HKU4-CoV−M^pro^ (PDB ID: 2YNB), and 45% similar with CoV-NL63 structure (PDB ID: 3TLO) (Figure 2A–D). Similarly, another target structure, SARS-CoV-2−RdRp (PDB ID: 7BW4) has 97% of structural similarities with previously reported SARS−CoV−RdRp structure (PDB ID: 6NUS), 18% of similar with enterovirus A71 (PDB ID: 3N6M), and 17% with human rhinovirus−RdRp (PDB ID: 1XR7). Then, using the protein sequence of the above PDB IDs constructed a phylogenic tree, followed by the Neighbor−Joining method. The phylogenic tree illustrated that the SARS-CoV-2−M^pro^ (relationship value, 0.025) and SARS-CoV-M^pro^ (relationship value, 0.020) were characteristically identical as presented in the same node/branch of the tree (Figure 2). Thus, the genomic diversity results suggest that the SARS-CoV-2 is a genetically modified strain of the CoV family and the mutated/modified genetic information may be helpful for drug development against SARS-CoV-2.

### 2.3. Virtual Screening by Molecular Docking Study

The present study used the PyRx 0.8 platform and AutoDock 4.2 software for molecular docking study against two SARS-CoV-2 targets (SARS-CoV-2−M^pro^ and -RdRp). Then, we have employed the BIOVIA-DSV-2019 software to visualize molecular interactions of protein−ligand complexes obtained from the docking study. The virtual screening was repeated twice to get the most errorless−cum−convenient protein−ligand binding energy or docking score (kcal/mol). Based on the docking score, the most potent six terpenoids were again docked manually in AutoDock to confirm the ligand’s crystallographic binding mode and appropriate docking score through 3-D and two−dimensional (2-D) molecular interactions with targets using the software BIOVIA-DSV-2019. Briefly, the ligand’s binding affinity with the target protein’s active side is measured as docking score by the number and type of interactions between ligand and amino acids residues of the functional binding pocket. Finally, we have selected the most potential marine terpenoids based on molecular docking score, types of interaction/bonding (hydrogen bond/ van der wall bond/pi−pi interaction, etc.), and the bond length (Å) with specific amino acid residues of the target protein. According to AutoDock software, a docking score in higher negative value exhibited ligand is considered the highest potency.

### 2.4. Physicochemical−Lipinski’s Rule of Five Profile Analyses

Lipinski’s rule of five (RO5) developed at Pfizer, states that a molecule should not exceed 500 g/mol in molecular weight (MW), the water-octanol of the partition coefficient (LogP) should be less than 5, the number of hydrogen donors (H-bd) groups <5, and the number of hydrogen acceptors (H-ba) groups <10. These parameters generally dictate the absorption and permeation of a drug. All criteria’s limitations are multiple of 5 (the name as rule of five) [69,70]. Introduced further restrictions regarding the polar surface area (PSA), the flexibility of molecules given by the number of rotatable bonds (PSA < 140 Å^2^ and number of rotatable bonds <10). The physicochemical features−standardized RO5 profiles of each marine terpenoid were recorded and analyzed using the bioinformatic tool, Swiss−ADME (http://www.swissadme.ch/; assessed on 12 February 2021) to predict the drug suitability profiles in comparison to reference drugs.

### 2.5. Toxicity and Pharmacokinetic Profile Prediction

The toxicity profile plays a vital role during the validation and acceptance of the human recommendation of any drug candidates. Several possible toxicity profiles, such as hepatotoxicity, carcinogenicity, immunotoxicity, mutagenicity, cytotoxicity, toxicity class, and lethal dose (LD_50_), were predicted using the ProTox tool (http://tox.charite.de/protox_II/; assessed on 12 February 2021). Several pharmacokinetic parameters such as blood−brain barrier (BBB), Gastrointestinal absorption (GI−abs.), P-glycoprotein, etc., and absorption, distribution, metabolism, excretion, and toxicity (ADME/T) profiles also recorded using SwissADME tool.

### 2.6. Overall Drug Likeness and SAR Analyses

The overall drug−likeness score or drug suitability value of terpenoids and reference drugs were predicted using the tool Molsoft (http://molsoft.com/mprop/; assessed on 24 February 2021), which guided further experimental validation of a chemical entity. Similarly, structural activity relationship (SAR) is a theoretical and versatile approach in the drug development module to know the chemical composition related to biological activity and possible inhibition mechanisms. Herein, the SAR analysis of potential terpenoids was analyzed and compared with reported antiviral IC_50_ (µM) values and docking scores (kcal/mol). This study used the ChemDraw Ultra 18.2 software to visualize the 2-D chemical structure of terpenoids during SAR analysis.

## 3. Results and Discussion

### 3.1. Virtual Screening by Molecular Docking Study

**Figure 3:**
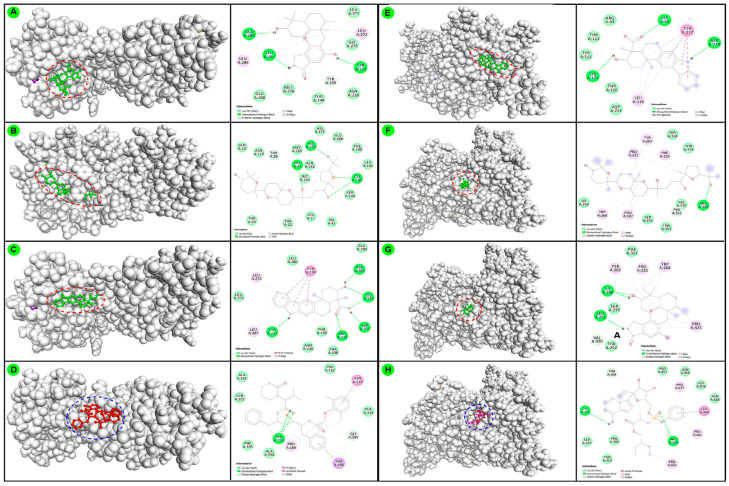
Graphical presentation of protein−ligand molecular interactions in the 3-D and 2-D arrangement of three most potential terpenoids against two different drug targets using the software, BIOVIA−DSV. The left side images, (**A**) Interaction of the stachyflin (−8.5 kcal/mol); (**B**) Interaction of thyrsiferol (−8.4 kcal/mol); (**C**) Interaction of xiamycin (−8.4 kcal/mol) and (**D**) Interaction of the reference drug, lopinavir (−8.4 kcal/mol) against SARS-CoV-2−M^pro^ (PDB ID: 6WTT); (**E**) Interaction of xiamycin (−9.3 kcal/mol); (**F**) Interaction of thyrsiferol (−9.2 kcal/mol); (**G**) Stachyflin (−8.8 kcal/mol); and (**H**) Interaction of the reference drug remdesivir (−7.4 kcal/mol) against SARS-CoV-2−RdRp (PDB ID: 7BW4), respectively.

The molecular docking scores (kcal/mol) from high−throughput virtual screening results of individual terpenoids and reference FDA−approved antiviral drugs against two drug targets are recorded (Table 2). Additionally, selected protein−ligand docking complex interactions were visualized (Figure 3). Based on the docking score, the brevione F (−8.4 kcal/mol), stachyflin (−8.4 kcal/mol), xiamycin (−8.4 kcal/mol), strongylin A (−8.3 kcal/mol), thyrsiferol (−8.2 kcal/mol), capillobenzofuranol (−8.0 kcal/mol), epitaondi−ol (−8.0 kcal/mol), and avarol (−7.9 kcal/mol) were some potential terpenoids that exhibited potency similar to the reference antiviral drugs, lopinavir (−8.4 kcal/mol) and darunavir (−7.5 kcal/mol) against SARS-CoV-2-M^pro^. Similarly, docking score of xiamycin (−9.3 kcal/mol), thyrsiferol (−9.2 kcal/mol), liouvilloside B (−8.9 kcal/mol), liouvilloside A (−8.8 kcal/mol), stachyflin (−8.7 kcal/mol), venustatriol (−8.7 kcal/mol), brevione F (−8.6 kcal/mol), thyrsiferyl−23−acetate (−8.6 kcal/mol) were some most potential terpenoids that exhibited higher docking score against SARS-CoV-2-RdRp, when compared with remdesivir (−7.4 kcal/mol) and favipiravir (−5.7 kcal/mol) the reference drugs (Figure 3). Overall, most terpenoids exhibited equivalent anti-CoV potency when compared with reference drugs. Thus, these potential terpenoids could be considered an alternative/ complementary therapeutic candidate for further experimental/pharmacological evaluation mainstream application against SARS-CoV-2.

Based on previous reports, the listed sixty−eight marine terpenoids showed antiviral activity against EV71, HCMV, HIV-1, H1N1, HSV-1, VSV, Rhinovirus, and CoV (see the reference in Table 1 for individual experimental study). Among these, several terpenoids IC_50_/ED_50_ value was <1 µM against some specific virus. For example, the stachyflin exhibited anti−H1N1 activity at IC_50_ = 0.003 µM; thyrsiferyl-23-acetate activity against HSV-1, CoV-A59 and VSV was within range of ED_50_ = 0.15–0.77 µM; thyrsiferol and venustatriol displayed potency against HSV-1, CoV-A59 and VSV within ED_50_ = 0.16–0.82 µM; avarol exhibited IC_50_ = 0.30 µM against HIV-1; aphidicolin exhibited IC_50_ = 0.59 µM against HSV, HCMV; mycaperoxide A exhibited IC_50_ = 0.6–4.0 µM against HSV-1 and VSV; mycaperoxide B exhibited IC_50_ = 0.58–2.35 µM against HSV-1, and VSV, solenolide A exhibited IC_50_ = 0.70 µM against rhinovirus and HSV; and spongiadiol exhibited IC_50_ = 0.75 µM against HSV-1, were recorded (Table 1). Specifically, five terpenoids namely halitunal, thyrsiferol, thyrsiferyl-23-acetate, reiswigin A, and venustatriol, were tested against CoV strains [34,44,55,62]. From a potential point of view, the current molecular docking results also verify and support the previous antiviral experimental records of terpenoids as exhibited similar types of efficacies against SARS-CoV-2 (Table 2).

Generally, terpenoids are more diverse groups of secondary metabolites that exhibited a broad−spectrum inhibition with multiple types of mode of action during the treatment against several diseases [71,72,73,74,75,76]. Most plant and marine terpenoids showed potent antiviral activity in cell lines; however actual mode of inhibition is still under investigation [77,78,79]. Especially, terpenoids showed virucidal activity against the various viruses by direct inactivation of free viral particles, avoiding host interaction, induced cell cycle arrest at G0 or G1 phase, and overall target to inhibit viral replication in host-cell [6,80,81]. In the SARS-COV-2 case, several terpenoids were displayed potent activity in different cell lines and mostly computational investigation without proper mode of inhibition. Hypothetically, terpenoids target to attach with angiotensin−converting enzyme-2 to avoid host−interaction and replication targeting M^pro^ and RdRp of SARS-COV-2 [6,80,82]. At this stage, more computational investigation, drug chemistry, and SAR analysis may help to explore more drug-action information. In addition, like virucidal activity, terpenoids showed potent antibacterial activity by disturbing membrane permeability and cell morphology, reducing salt tolerance, inhibiting biofilm pathways, and targeting the cell membrane synthesis pathways [75,83]. Similarly, it showed potential antiparasitic/antimalarial activity by binding with hemin part of infected erythrocytes like the mainstream chloroquine [73,84], anticancer by disruption of microtubules, suppress the cyclin D1, apoptosis, and cell cycle arrest [74,85], treat neuro−disorder by inhibiting the neurotransmitters GABA and cannabinoid receptor-2 [86,87], antidiabetic potency by activating the α-adrenoreceptors to increase the release of an opioid peptide β-endorphin [72,73,88].

On the other hand, the CADD is an ideal platform in the current drug development module to accelerate the ‘lead’ selection by a target−specific approach. [26,66]. Molecular docking mainly expresses a candidate’s biological potential against a particular target enzyme/protein associated with disease initiation and proliferation by molecular interaction/binding affinity [26,66]. However, sometimes molecular docking produces ambiguous results; as indicated, proper ligand and protein structure analysis should follow to get proper outputs. For example, Hosseini et al. performed a docking against M^pro^ with PDB ID: 6LU7_A (similar to our target M^pro^ PDB ID: 6WTT) with several standard drugs. From which, the lopinavir displayed three docking scores: −7.26 from the Glide, −9.3 from AutoDock Vina, and −81.57 from rDock software [89]. Thus, docking programs/software are algorithm-specific, represented in different patterns from software to software. For example, AutoDock, ArgusLab, Glide represents in negative sign, while Hex, PathDock, HDOCK like docking program represents in positive sign. In addition, there is no standard range of docking score or potentiality has been established. The only comparison between desire ligands and stand drugs of docking score are the ideal option to select the best potential candidates from many—still, several improvements in molecular docking study under investigation and hopefully shoutout this issue soon. Indeed, including docking score and several other predictions such as physicochemical, cytotoxicity, drug−likeness, and pharmacokinetics profiles through various computational tools also help select the most potential lead chemicals. Molecular docking, like other computational-based drug analyses, helps reduce time and experiments during the selection of potential candidates based on training set candidates. Overall, experimental/clinical work on compounds’ activity, toxicity, and pharmacokinetics results is the actual validator for mainstream drug use [33,48,49].

### 3.2. Physicochemical−Lipinski’s Rule of Five Profile Analyses

The physicochemical property or the well recommended RO5 properties of sixty-eight marine terpenoids are summarized in Table S1. Based on the standard RO5, most of the marine terpenoids obey the RO5 rule. Briefly, the RO5 violation occurs when the chemical’s molecular weight is more than 500 g/mol, XLogP value more than five, H−bond accepter number more than 10, and the tPSA value more than 142 Å [23,69,70]. Broadly, eleven compounds violate based on molecular weight, twenty-one violates XlogP values, two disrupt the H-bond acceptors and donors’ suitable drug-able values mentioned in the RO5. In addition, except for the most potential candidate, the stachyflin and other marine products slightly violate one or two parameters of RO5. Besides, three drugs (darunavir, lopinavir, and remdesivir) from four reference drugs also violate the RO5 rules. Thus, physicochemical and RO5 profile prediction and analysis to select potential drug-able oral drug candidates at the early or preclinical stage are ideal approaches and mostly accessible through bioinformatic and chemoinformatic tools.

### 3.3. Toxicity and Pharmacokinetic Profile Prediction

The computationally predicted hepatotoxicity, carcinogenicity, immunotoxicity, mutagenicity, and cytotoxicity profiles of marine compounds and referral drugs (kg/body weight) are recorded (Table 2). This type of prediction is statistical reports of previously registered training set compound in the ProTox tool. This toxicity data confirmed that most marine compounds are safe related to their hepatotoxic, carcinogenic, and mutagenic profiles, as indicated in green and light green. However, some issues with immunotoxicity are marked in red and pale pink [90,91]. For example, the selected drug stachyflin with toxicity class five (V) indicated a non−toxic/safer chemical. According to toxicity class, higher toxicity class is considered to be higher in safety. Out of sixty-eight marine terpenoids, fifteen compounds were under the toxicity class of V-toxicity. Besides five terpenoids (in class-I and -II), most terpenoids displayed under class-III and IV from toxicity class as indicated as minor in toxicity. Resultant data reveals the reference drug lopinavir to be safer than other drugs. This study claims marine terpenoids to be safer based on their high LD_50_ value and toxicity concentration. Thus, toxicity prediction through the computational tool offers the advantages of determining highly toxic compounds early, and can reduce experimental and animal toxicity studies.

Similarly, computational tools can predict possible ADMET properties for a chemical according to the training set chemical present in the system. The ADMET properties of all marine terpenoids and reference drugs are recorded in Table S2. Only eight terpenoids and reference drug darunavir and favipiravir showed low gastrointestinal absorption properties. Similarly, thirty-four terpenoids with all three drugs (except favipiravir) manifest strength to cross the blood-brain barrier. Additionally, CYP1A2, CYP2C19, CYP2C9, CP2D6, and CYP3A4 inhibition activity also predict an average condition as consumption/loss of drug concentration before target inhibition inside the body. The skin permeability (LogKp) value of terpenoids is similar to the reference drugs [69]. According to the tool, SwissADME, more negative LogKp values indicated lower permeability as LogKp depends on the molecular size and lipophilicity of a compound. The predicted ADMET properties of terpenoids were similar to the reference drugs, which gives a positive indication to develop a potential drug. Generally, ADMET properties can be consistently measured in mice models in the end session of the drug development. Nevertheless, prediction with computational tools is also beneficial to know the probable information, and that could be helpful to reduce the resource and *in vivo* experiments in current drug development [92].

### 3.4. Overall Drug Likeness and SAR Analyses

The drug-likeness or drug suitability score associated with the physiological, pharmacokinetics, and toxicity profile was also predicted (Table 2). From the drug-likeness record, twenty-eight terpenoids showed positive drug-likeness with a range of 0.03–1.08, while the compound seco−pseudopterosin I displayed the most favorable score of 1.08. Terpenoids namely, avarol (0.06), bromophycolide A (0.28), capillobenzofuranol (0.29), liouvilloside A (0.50), liouvilloside B (0.45), stachyflin (0.34), strongylin A (0.39) exhibited the positive drug-likeness scores. On the other hand, epitaondiol (−0.41), thyrsiferol (−0.33), thyrsiferyl-23-acetate (−0.40), venustatriol (−0.33), and xiamycin (−0.02) exhibited negative drug−likeness scores. Overall, terpenoids such as acDa-1, pseudopterosin P, seco-pseudopterosin I, usneoidol E, and usneoidol Z were selective compounds possessing drug suitability profiles, active docking score, and safer toxicity classes. Similarly, lopinavir also showed 1.10, while the favipiravir exhibited −0.87. According to the molsoft tool, the drug−likeness between >0 and <2 is a suitable score for a chemical. Thus, the computational investigation indicated that some selective terpenoids have enough potential profiles to convert ‘lead’ to ‘drug’. Mostly, the CADD is a potential and cost-effective tool in current drug discovery and even pharmaceutical companies during lead drug selection.

**Figure 4:**
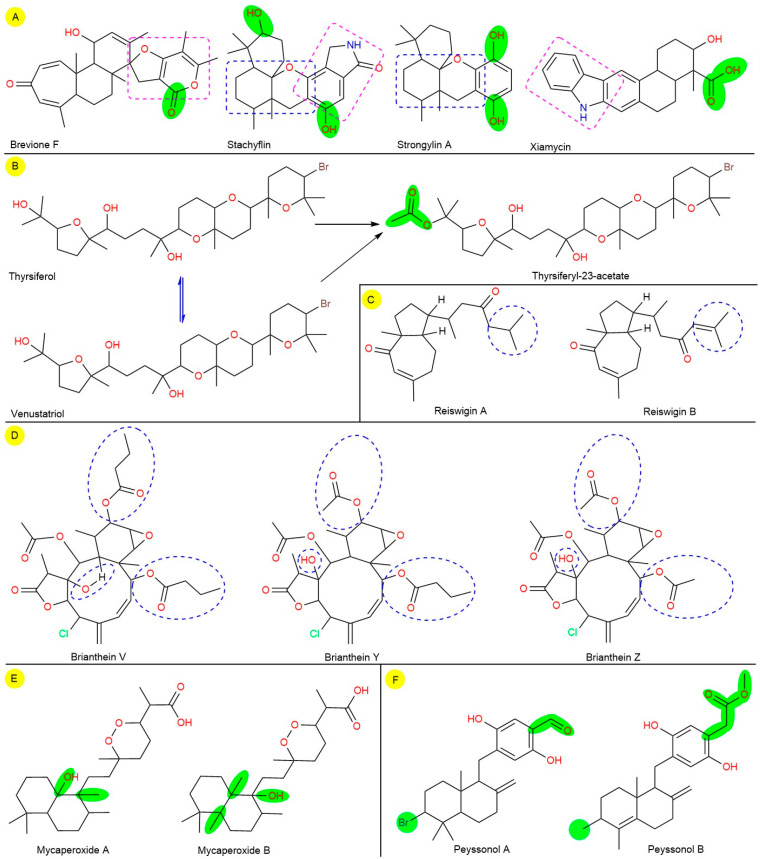
A schematic presentation during the structural−activity relationship (SAR) on various sets (**A**–**F**) of marine terpenoids. The chemical structures were generated using the software ChemDraw 18.2 Ultra.

From SAR analysis, brevione F, stachyflin, strongylin A, and xiamycin terpenoids contain some common pharmacophores, where the hydroxy (-OH) group differentiates them within reported antiviral activity and current docking score (Figure 4A). For example, thyrsiferol exhibited antiviral activity within ED_50_ = 0.16–0.82 µM, and thyrsiferyl-23-acetate exhibited antiviral activity within ED_50_ = 0.15–0.77 µM against HSV-1, CoV-A59, and VSV. Similarly, thyrsiferol showed the docking scores, −8.3 and −7.4; and thyrsiferyl-23-acetate showed −8.2 and −9.2 kcal/mol against two targets of SARS-CoV-2, respectively. Furthermore, the SAR analysis revealed that a slight variation in recorded experimental and computational results between both compounds is due to the acetaldehyde group (CH_3_CHO) in thyrsiferyl-23-acetate structure as well showed comparatively less activity than thyrsiferol (Figure 4B). Another pair of broad−spectrum antiviral marine diterpenes, reiswigin A and B from marine sponge (*Epipolasis reiswigi*), exhibited potential activity against HSV-1 and VSV with an EC_50_ value, 6.57 and 6.62 μM, respectively. In addition, both diterpenes exited their potency against CoV-A59 strain with ten times higher EC_50_ value, 65. 7 and 66.2 μM [55]. According to SAR analysis, both chemical structures are slightly different by attachment of isobutane in reiswigin A and isobutene reiswigin B at the tail end (Figure 4C). The attachment of isobutene mostly slightly reduces the antiviral activity. From computational results, a similar docking score against SARS-CoV-M^pro^, non−toxic profiles (class-V and IV) with negative drug-likeness scores, −0.28 and −0.11 (Table 2).

On the other hand, the polyfunctional chlorine attached diterpenoids, brianthein-V, -Y, and -Z from *Briarurn asbestinum* (a marine soft coral), displayed anti-CoV against CoV−A59 strain with higher EC_50_ values, 83.75, 702.98, and 147.87 μM, respectively [34]. From SAR analysis, cyclodecene (C_10_H_18_), cyclohexene oxide (C_6_H_10_O), and γ-butyrolactone or dihydrofuran-2(3H)-one (C_4_H_6_O_2_) with chlorine in each in all three structures. Indeed, the presence of two methyl butyrate, one methyl acetate with a methyl−like group attachment within cyclodecene ring in brianthein-V, one methyl butyrate, two methyl acetate with a hydroxy group attachment with cyclodecene ring in brianthein-Y, and three methyl acetate with a hydroxy group attachment with cyclodecene ring differentiate the structure and activity too (Figure 4D). Similarly, brianthein-V showed a higher docking score, −7.0 kcal/mol against SARS-CoV-M^pro^, and brianthein-V showed a higher docking score, −7.5 kcal/mol against SARS-CoV-2−RdRp. However, brianthein-Y potential positive drug-likeness score, 0.29, while the other two derivatives showed negative scores, −0.77 and −0.99, with the same toxicity class-II and LD_50_ value 7 mg/kg. Overall, the assembly of all functional groups in all three structures with slight variation showed poor activity with higher cytotoxicity, which may be due to presence of two corrosive/toxic functional groups, cyclohexene oxide and γ-butyrolactone with a chlorine atom.

Another pair of cyclic norsesterterpene peroxides, mycaperoxide A and B from marine sponge (*Mycale* sp.), exhibited potential antiviral activity against HSV-1 and VSV with IC_50_ values, 0.6–4.0 and 0.58–2.35 μM, respectively [51]. According to SAR, both structures are nearly equal with an extra -CH_3_ group, and the position -OH is changed mycaperoxide B as the chemical formula C_24_H_42_O_5_ and C_25_H_44_O_5_. However, the presence of additional -CH_3_ group and position of -OH group comparatively higher the antiviral activity (Figure 4E). From computational investigation also showed similar types of results as slightly varies in molecular docking score with similar LD_50_, bioavailability, and toxicity classes. Nevertheless, both chemicals showed a negative drug-ability score, −0.25 and −0.69. In addition, peyssonol A and B are another pair of sesquiterpene hydroquinones derived from marine red alga (*Peyssonnelia* sp.) and exhibited anti−HIV (HIV-1 and HIV-2) activity [52]. Combatively, peyssonol B displayed potential activity against HIV-1 at an IC_50_ value, 4.30 μM, and against HIV-2 at 14.70 μM than peyssonol A at 6.40 21.30 μM, respectively [52]. The SAR analysis also confirmed that the presented an acetaldehyde and bromine atom in peyssonol A and peyssonol B structure contains a double bond in parent decahydronaphthalene nucleus, a methyl propionate in place of acetaldehyde, and no bromine atom was present. Overall, the presence of single methyl propionate comparatively showed higher anti-HIV activity than acetaldehyde and bromine combination (Figure 4F). Similarly, peyssonol B showed a higher docking score, −7.5 kcal/mol against SARS-CoV-M^pro^, with a positive drug-ability, 0.09 under toxicity class−IV. On the other hand, peyssonol A showed a higher docking score, −7.3 kcal/mol against SARS-CoV-2−RdRp, a negative drug-ability, −0.48 with higher non-toxic than peyssonol B with toxicity class-V (Table 2).

Overall, the SAR analysis confirmed that small attachment in the parent scaffold directly influenced the biological activity, toxicity, and pharmacokinetics. Thus, during drug development, SAR analysis also plays an essential role in biological activity and toxicity profiles analysis of a series of drug candidates based on their chemical structure [34,51,52,55,93,94,95].

The evolution of zoonotic viruses H1N1, Nipah, Ebola, and present SARS-CoV-2 was grown in any conditions followed the ‘theory of existence’ with a newer genetic arrangement through drug pressure or any environmental impacts [96,97]. Indeed, human involvement in other ecosystems out of ethics for their benefits may be another factor for developing such shocking outbreaks worldwide. The newly emerging SARS-CoV-2 is a novel and aggressive\e pathogen with the rapid development of newer variants and transmission [2,3]. The global research community already spent enormous effort on its genome study to get more information on evolution and pathogenesis and continue investigating active drug/vaccine candidates to control the pandemic. The study of genomic diversity gives a few inherited characteristics that are most beneficial for locating potential therapeutic candidates. After enormous efforts, some vaccine candidates are used without FDA approval to control the emerging situation. However, the rapid genetic mutations reduce the efficacy of proposed drug and vaccine candidates. Thus, success against such a nasty virus is a challenge in front of the global scientific community.

In the meantime, worldwide laboratories share a massive amount of genomic information, which is most difficult to analyze without bioinformatics tools. Additionally, project many potential drugs and natural products against SARS-CoV-2 with the CADD platform [18,79,98,99,100]. At present, several tools and databases of bioinformatics are available for gene analysis to vaccine/drug development. Mostly, systematic computational screening is the most cost−effective and resource-saving approach towards select the ‘lead’ candidate(s) in the current drug development module [18,23,89,101]. Overall, bioinformatics tools continuously explore hidden genomic information and mainly identified potential natural lead candidates from different sources against SARS-CoV-2 for further experimental study and validation [14,100,102].

Generally, natural resources are the most conservative sources for therapeutic purposes since the primitive age. The toxicity, pharmacokinetics, and drug-likeness profiles are the most crucial parameters for selecting potential candidates. Thus, bioinformatics tools could be considered the most resource-saving approach to predicting drug−ability activities early stage. The standardized RO5 rule based on physiological properties, toxicity prediction, ADMET, and overall drug-likeness property analysis finds the drawback of any selected candidates [69,103,104]. Mainly, toxicity profiles depend on the drug’s chemical composition, while withdrawal decisions depending on its biological activity concentration. Interestingly this study confirms that selected terpenoids exhibits antiviral activity in lower concentrations while delivers no toxicity even after 10-fold concentration. On the other hand, several alternative techniques such as structural modification, chemical conjugation, nano-drug delivery, and polymer-coated formulations with tools of medicinal chemistry are available to improve the drug-ability profiles of most potential candidates [95,105,106].Overall, the above computational investigations with various bioinformatic, chemoinformatic tools and perspective drug analysis by SAR can analyze the potency, drug chemistry, and drug−ability to select potential ‘lead’ candidates from a plethora of broad-spectrum antiviral marine terpenoids, i.e., brevione F, stachyflin, and xiamycin systematically and cost−effectively to counter the newly emerged SARS-CoV-2.

## 4. Conclusions

Currently, the global primary health system suffers from emerging SARS-CoV-2 or COVID-19, and there are urgent necessities of potential regimens to control the gruesome virus. Including drug repurposing, several natural products have been under various experimental/clinical validation stages towards the control of SARS-CoV-2. Opportunistically, locate the most potential drug ‘lead’ candidates against SARS-CoV-2 from a broad-spectrum antiviral marine terpenoids library through bioinformatics, cheminformatics, and medicinal chemistry tools may consider as a prudent approach in the current drug development module. From an extensive computational drug development point of view, brevione F, liouvillosides A and B, strongylin A, stachyflin, thyrsiferol, thyrsiferyl-23-acetate, and xiamycin are some potential terpenoids based on strong binding−affinity with SARS-CoV-2−M^pro^ and -RdRp enzymes and ideal drug−ability profiles comparatively higher than the reference antiviral drugs (darunavir, favipiravir, lopinavir, and remdesivir). Overall, docking score, drug-likeness, and toxicity profile concluded that the stachyflin is the most potent, drug−able, and safe candidate that could use against SARS-CoV-2 after some pharmacological validation.

## Figures and Tables

**Table 1:** Selected sixty−eight marine terpenoids with broad−spectrum antiviral potency against different viral strains.

Sl. No.	Name of the Terpenoid (Terpenoid Type)	Marine Source (Organism Type)	Antiviral Activity	Recorded IC_50_/EC_50_ (µM)	References
1.	AcDa-1 (Diterpene)	*Dictyota menstrualis* (Algae)	HIV-1	IC_50_: 35–70	[27]
2.	Acetyl ehrenberoxide B (Diterpenoid)	*Sarcophyton ehrenbergi* (Soft coral)	HCMV	EC_50_:22.0	[28]
3.	Alismol (Sesquiterpenoid)	*Litophyton arboretum* (Soft coral)	HIV-1	IC_50_:7.20	[29]
4.	Aphidicolin (Diterpene)	*Cephalosporium aphidicola* (Sponge)	HSV, HCMV	IC_50_: 0.59	[30]
5.	Atomaric acid (Meroditerpenoid)	*Stypopodium zonale* (Brown algae)	HMPV	IC_50_: 7.96	[31]
6.	Avarol (Sesquiterpene hydroquinone)	*Dysidea avara* (Sponge)	HIV-1	IC_50_: 0.30	[32]
7.	Brevione F (Spiroditerpenoid)	*Penicillium* sp. (Fungus)	HIV-1	EC_50_: 14.7	[33]
8.	Brianthein V (Diterpene)	*Briarurn asbestinum* (Soft coral)	CoV-A59	EC_50_: 83.75	[34]
9.	Brianthein Y (Diterpene)	*Briarurn asbestinum* (Soft coral)	CoV-A59	EC_50_: 702.98	[34]
10.	Brianthein Z (Diterpene)	*Briarurn asbestinum* (Soft coral)	HSV-1 CoV-A59	EC_50_: 147.87	[34]
11.	Bromophycolide A (Diterpene benzoate)	*Callophycus serratus* (Red algae)	HIV-1	IC_50_: 9.1–9.8	[34]
12.	Capillobenzofuranol (Furano−sesquiterpenoid)	*Sinularia capillosa* (Soft coral)	HCMV	EC_50_: 13.5	[35]
13.	Capilloquinol (Diterpene quinoid)	*Sinularia capillosa* (Soft coral)	HCMV	ED_50_ 11.24	[36]
14.	Curcuphenol (Sequiterpene)	*Didiscus axeata* (Sponge)	HCV	EC_50_: 31.2	[37]
15.	Cyanthiwigin B (Diterpene)	*Myrmekoderma styx* (Sponge)	HIV-1 HBV	EC_50_: 42.1 EC_50_: >333.33	[38]
16.	Da-1 (Diterpene)	*Dictyota menstrualis* (Brown algae)	HIV-1	IC_50_:10−40	[27]
17.	Dehydrofurodendin (Sesterterpene)	*Lendenfeldia* sp. (Sponge)	HIV-1	IC_50_: 3.2−5.6	[39]
18.	Dolabelladienol A (Diterpene)	*Dictyota pfaffii* (Brown algae)	HIV-1	EC_50_: 2.9 ± 0.2	[40]
19.	Dolabelladienol B (Diterpene)	*Dictyota pfaffii* (Brown algae)	HIV-1	EC_50_: 4.10	[40]
20.	Ehrenbergol A (Diterpenoid)	*Sarcophyton ehrenbergi* (Soft coral)	HCMV	IC_50_: 132.1	[41]
21.	Ehrenbergol B (Diterpenoid)	*Sarcophyton ehrenbergi* (Soft coral)	HCMV	IC_50_: 13.73	[41]
22.	Ehrenbergol C (Diterpenoid)	*Sarcophyton ehrenbergi* (Soft coral)	HCMV	EC_50_: 52.91	[28]
23.	Epispongiadiol (Diterpene)	*Spongia* sp. (Sponge)	HSV-1	IC_50_: 37.65	[42]
24.	Epitaondiol (Meroditerpenoid)	*Stypopodium zonale* (Brown algae)	HMPV	IC_50_: 2.45	[31]
25.	Erythro-N-dodecanoyl-doco sasphinga-(4E,8E)-dienine (Sesquiterpenoid)	*Litophyton arboreum* (Soft coral)	HIV-1	IC_50_: 4.80	[43]
26.	Gyrosanol A (Diterpenoid)	*Sinularia gyrosa* (Soft coral)	HCMV	IC_50_: 2.60	[35]
27.	Gyrosanol C (Diterpenoid)	*Sinularia gyrosa* (Soft coral)	HCMV	IC_50_: 3.70	[35]
28.	Halitunal (Diterpene aldehyde)	*Halimeda tuna* (Green seaweed)	CoV-A59	IC_50_: 56.49	[44]
29.	Helioporin A (Diterpene)	*Heliopora coerulea* (Blue coral)	HSV-1	IC_50_: 12.19	[45]
30.	Helioporin B (Diterpene)	*Heliopora coerulea* (Blue coral)	HSV-1	IC_50_: 4.84	[45]
31.	Hyrtiosal (Sesterterpenoid)	*Hyrtios erectus* (Sponge)	HIV-1	IC_50_: 9.60	[46]
32.	Ilimaquinone (Sesquiterpenoid quinones)	*Dysudea arenaria* (Sponge)	HIV-1	IC_50_: 16.4	[47]
33.	Integric acid (Sesquiterpenoid)	*Xylaria* sp. (Ascomycetous fungi)	HIV-1	IC_50_: 5−20	[48]
34.	Isospongiadiol (Diterpene)	*Spongia* sp. (Sponge)	HSV-1	IC_50_: 6.02	[42]
35.	Liouvilloside A (Trisulfated triterpene glycoside)	*Staurocucumis liouvillei* (Sea cucumber)	HSV-1	IC_50_: 6.96	[49]
36.	Liouvilloside B (Trisulfated triterpene glycoside)	*Staurocucumis liouvillei* (Sea cucumber)	HSV-1	IC_50_: 6.93	[49]
37.	Lobohedleolide (Cembrane diterpenoid)	*Lobophytum* sp. (Soft coral)	HIV-1	IC_50_: 10.90	[50]
38.	Mycaperoxide A (Norsesterterpene)	*Mycale* sp. (Sponge)	HSV-1, VSV	IC_50_: 0.6−4.0	[51]
39.	Mycaperoxide B (Norsesterterpene)	*Mycale* sp. (Sponge)	HSV-1, VSV	IC_50_: 0.58−2.35	[51]
40.	Peyssonol A (Sesquiterpene hydroquinone)	*Peyssonnelia* sp. (Red algae)	HIV-1 HIV-2	IC_50_: 6.40 IC_50_: 21.30	[52]
41.	Peyssonol B (Sesquiterpene hydroquinone)	*Peyssonnelia* sp. (Red algae)	HIV-1 HIV-2	IC_50_: 4.30 IC_50_: 14.70	[52]
42.	Pseudopterosin P (Diterpene glycoside)	*Pseudopterogorgia elisabethae* (Sponge)	HSV-1 & VZV, HCMV	EC_50_: 2.90 EC_50_: 2.60	[53]
43.	Puupehedione (Shikimatesequiterpene)	*Verongida* and *Dictyoceratida* sp. (Sponge)	HIV-1	IC_50_: 3.01	[54]
44.	Reiswigin A (Diterpene)	*Epipolasis reiswigi* (Sponge)	HSV-1 & VSV CoV-A59	EC_50_: 6.57 EC_50_: 65.7	[55]
45.	Reiswigin B (Diterpene)	*Epipolasis reiswigi* (Sponge)	HSV-1 & VSV, CoV-A59	EC_50_: 6.62 EC_50_: 66.22	[55]
46.	Rietone (Triprenylhydro−quinone)	*Alcyonium fauri* (Soft coral)	HIV-1	IC_50_:9.32	[56]
47.	Secopseudopterosin H (Diterpene glycoside)	*Pseudopterogorgia elisabethae* (Sponge)	HSV-1, VZV & HCMV	IC_50_: >10	[53]
48.	Seco−pseudopterosin I (Diterpene glycoside)	*Pseudopterogorgia elisabethae* (Sponge)	HSV-1, VZV & HCMV	IC_50_: >10	[53]
49.	Secocrassumol (Seco−cembranoid diterpene)	*Lobophytum crissum* (Soft coral)	HCMV	IC_50_: 12.69	[43]
50.	Sinuleptolide (Diterpene)	*Sinulariana nolobata* (Soft coral)	HCMV	ED_50_: 5.51	[57]
51.	Smenospongine (Sesquiterpenoid quinone)	*Dysudea arenaria* (Sponge)	HIV-1	IC_50_: 176.1	[47]
52.	Smenotronic acid (Sesquiterpenoid quinone)	*Dysudea arenaria* (Sponge)	HIV-1	IC_50_: 130.4	[47]
53.	Solenolide A (Diterpenoid lactone)	*Solenopodium* sp. (Marine octocoral)	Rhinovirus, HSV	IC_50_: 0.70	[58]
54.	Solenolide E (Diterpenoid lactone)	*Solenopodium* sp. (Marine octocoral)	Rhinovirus, HSV	IC_50_: 28.40	[58]
55.	Spongiadiol (Furanoditerpene)	*Spongia* sp. (Sponge)	HSV-1	IC_50_: 0.75	[42]
56.	Stachybogrisephenone B (Sesquiterpenoid)	*Stachybotry* sp. HH1 ZSDS1F1-2 (Sponge)	EV71	IC_50_: 30.10	[59]
57.	Stachyflin (Terpenoid)	*Stachybotrys* sp. RF−7260 (Fungus)	H1N1	IC_50_: 0.003	[60]
58.	Strongylin A (Sesquiterpene hydroquinone)	*Strongylophor ahartmani* (Sponge)	H1N1	IC_50_: 18.89	[61]
59.	Thyrsiferol (Triterpene)	*Laurencia venusta* (Red algae)	HSV-1, CoV−A59, VSV	ED_50_: 0.16–0.82	[62]
60.	Thyrsiferyl-23-acetate (Triterpene)	*Laurencia venusta* (Red algae)	HSV-1, CoV-A59, VSV	ED_50_: 0.15–0.77	[62]
61.	Usneoidol E (Meroterpene)	*Cystoseira usneoides* (Brown seaweed)	HSV, VSV	IC_50_: 7.62 IC_50_: 7.83	[63]
62.	Usneoidol Z (Meroterpene)	*Cystoseira usneoides* (Brown seaweed)	HSV, VSV	IC_50_: 8.47 IC_50_: 13.13	[63]
63.	Venustatriol (Triterpene)	*Laurencia venusta* (Red algae)	HSV-1, CoV-A59, VSV	ED_50_: 0.16–0.82	[62]
64.	Xiamycin (Pentacyclic indolosesquiterpene)	*Bruguiera gymnorrhiza* (Marine mangrove bacteria)	HIV	IC_50_: 5–20	[64]
65.	7β-acetoxy-24-methyl cholesta-5-24(28)-diene-3,19-diol (Sesquiterpenoid)	*Litophyton arboreum* (Soft coral)	HIV-1	IC_50_: 4.85	[43]
66.	(7Z)-Lobohedleolide (Cembranoid diterpene)	*Lobophytum* sp. (Soft coral)	HIV-1	EC_50_: 13.93	[50]
67.	8,10,18-trihydroxy-2,6-dolabelladiene (Diterpene)	*Dictyota pfaffii* (Brown algae)	HSV-1	EC_50_: 5.10	[65]
68.	17-dimethyl amino-lobohedleolide (Cembranoid diterpene)	*Lobophytum* sp. (Soft coral)	HIV-1	IC_50_: 8.80	[50]

CoV-19, coronavirus-19; EV71, antienterovirus 71; H1N1, influenza subtype H1N1 or swine flu, HCMV, human cytomegalovirus; HIV-1, human immune deficiency virus-1; HMPV, human metapneumovirus; HSV-1, herpes simplex-1 virus; VSV, vesicular stomatitis virus.

**Table 2:** Recorded individual docking scores (kcal/mol) against SARS-CoV-2 M^pro^ and RdRp, drug-likeness, lethal dose (kg/mg), bioavailability score, toxicity profiles, toxic class, using the tools, PyRx, Molsoft and ProTox, respectively.

Sl. No.	Docking Score		Drug Likeness Score	LD_50_ Score (mg/kg)	BA Score	Toxicity Profiles					
	M^pro^*/*3CL^pro^	RdRp				HT	CG	IT	MG	CT	TC
1.	−7.4	−6.7	0.42	1400	0.55	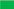	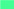	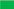	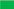	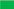	IV
2.	−7.0	−7.1	−1.02	2000	0.55	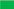	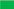	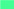	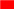	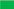	IV
3.	−6.5	−7.0	−1.03	1200	0.55	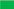	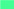	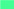	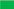	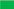	IV
4.	−6.8	−6.9	−0.15	500	0.55	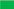	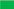	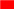	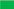	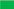	IV
5.	−7.8	−7.1	0.58	750	0.56	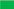	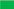	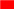	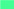	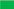	IV
6.	−7.9	−7.5	0.41	2300	0.55	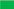	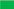	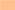	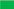	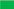	V
7.	−8.4	−8.6	0.06	5	0.55	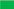	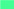	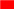	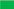	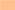	I
8.	−6.5	−7.5	−0.77	7	0.55	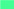	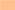	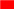	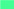	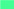	II
9.	−6.6	−7.0	0.29	7	0.55	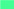	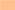	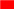	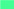	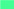	II
10.	−7.0	−6.6	−0.99	7	0.55	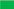	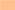	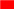	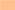	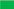	II
11.	−7.4	−8.0	0.28	1000	0.17	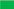	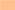	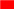	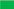	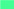	IV
12.	−8.0	−7.3	0.29	500	0.55	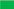	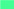	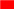	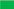	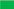	IV
13.	−7.7	−7.7	−0.75	500	0.55	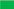	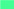	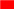	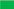	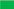	IV
14.	−5.4	−6.1	0.28	1500	0.55	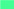	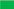	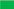	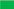	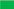	IV
15.	−7.2	−7.1	0.03	2200	0.55	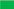	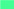	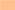	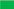	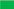	V
16.	−6.4	−6.2	0.20	750	0.55	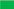	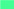	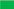	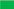	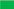	IV
17.	−7.5	−5.7	−1.14	600	0.55	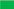	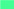	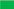	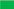	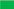	IV
18.	−6.8	−7.0	−0.17	5000	0.55	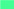	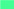	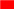	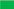	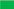	V
19.	−6.7	−6.8	−0.17	5000	0.55	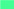	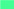	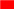	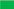	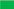	V
20.	−3.4	−3.3	−1.28	500	0.55	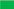	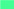	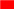	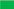	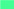	III
21.	−2.9	−3.5	−0.41	500	0.55	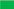	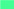	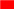	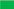	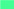	III
22.	−3.0	−3.2	−0.41	500	0.55	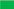	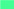	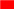	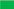	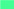	III
23.	−6.7	−7.0	−0.34	79	0.55	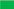	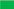	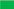	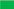	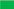	III
24.	−8.0	−8.0	−0.41	1000	0.55	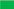	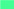	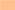	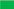	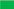	IV
25.	−6.8	−7.7	−1.08	2000	0.55	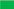	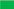	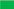	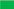	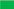	IV
26.	−7.1	−7.9	−0.10	1016	0.55	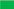	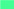	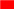	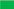	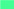	IV
27.	−6.1	−7.0	−0.81	2000	0.55	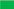	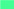	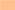	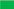	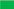	IV
28.	−6.5	−6.8	−0.54	950	0.56	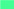	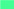	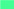	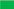	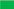	IV
29.	−7.8	−7.5	−0.78	500	0.55	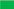	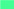	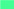	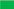	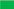	IV
30.	−6.5	−7.7	−0.63	4000	0.55	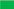	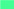	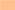	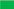	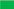	V
31.	−7.8	−7.8	−0.29	79	0.55	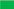	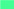	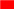	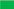	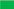	III
32.	−7.3	−7.2	0.10	2800	0.56	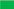	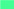	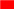	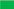	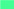	V
33.	−6.7	−6.5	0.25	5000	0.56	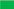	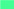	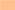	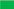	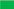	IV
34.	−7.1	−7.1	−0.25	79	0.55	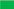	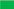	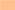	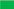	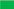	III
35.	−7.3	−8.8	0.50	4000	0.17	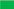	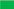	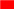	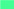	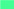	V
36.	−7.5	−8.9	0.45	4000	0.17	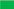	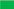	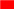	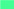	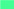	V
37.	−6.7	−7.3	−1.40	1000	0.56	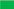	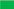	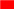	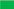	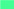	IV
38.	−6.9	−7.5	−0.25	10000	0.56	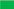	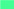	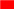	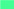	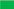	VI
39.	−6.4	−6.8	−0.69	10000	0.55	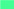	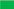	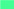	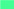	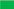	VI
40.	−6.3	−7.3	−0.48	2300	0.55	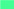	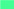	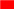	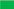	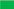	V
41.	−7.5	−7.1	0.09	860	0.55	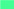	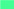	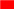	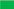	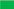	IV
42.	−7.7	−7.6	0.56	3000	0.55	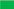	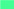	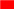	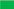	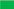	V
43.	−7.8	−7.9	−0.47	105	0.56	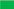	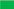	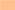	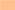	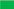	III
44.	−6.1	−6.5	−0.28	5000	0.55	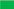	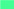	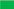	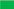	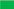	V
45.	−6.1	−6.0	−0.11	400	0.55	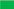	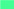	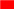	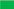	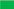	IV
46.	−6.1	−7.4	−0.32	400	0.55	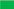	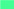	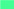	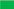	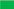	IV
47.	−7.4	−7.3	0.29	3000	0.55	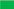	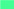	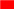	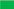	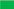	V
48.	−6.9	−7.5	1.08	3000	0.55	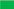	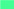	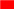	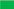	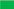	V
49.	−7.1	−6.4	0.41	3000	0.55	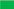	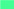	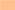	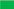	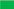	IV
50.	−8.0	−7.5	−1.12	1000	0.55	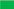	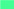	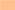	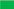	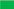	IV
51.	−7.2	−7.5	0.29	100	0.56	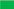	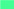	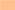	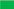	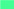	III
52.	−7.2	−7.0	−0.07	8	0.56	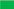	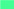	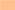	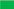	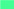	II
53.	−6.5	−7.0	−0.40	7	0.55	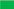	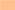	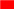	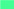	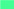	II
54.	−7.5	−7.3	−0.27	750	0.55	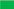	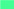	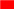	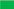	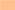	III
55.	−7.5	−7.3	−0.34	79	0.55	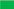	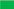	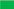	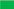	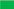	III
56.	−6.0	−6.4	−0.27	3200	0.55	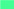	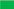	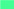	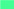	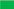	V
57.	−8.4	−8.7	0.34	4000	0.55	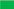	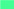	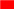	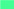	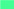	V
58.	−8.3	−7.4	0.39	1000	0.55	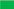	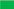	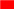	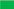	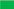	IV
59.	−8.2	−9.2	−0.33	2600	0.55	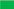	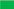	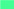	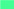	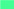	V
60.	−8.0	−8.6	−0.40	5000	0.55	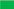	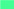	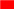	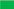	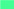	V
61.	−6.7	−6.2	0.47	2000	0.55	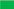	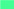	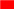	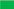	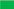	IV
62.	−6.3	−6.7	0.47	2000	0.55	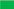	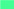	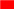	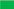	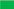	IV
63.	−7.6	−8.7	−0.33	2600	0.56	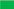	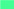	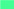	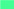	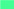	V
64.	−8.4	−9.3	−0.02	1000	0.56	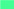	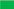	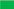	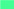	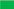	IV
65.	−6.8	−7.8	−0.08	2000	0.55	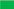	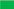	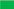	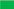	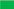	IV
66.	−7.3	−7.6	−0.84	1200	0.56	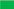	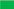	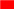	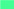	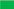	IV
67.	−7.6	−6.9	−0.04	5000	0.55	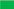	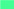	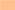	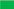	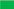	IV
68.	−6.6	−7.0	−0.25	1000	0.55	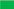	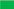	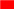	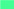	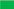	IV
69.	−7.5	ND	0.60	245	0.55	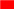	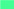	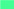	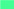	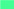	III
70.	−8.4	ND	1.10	5000	0.55	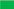	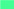	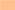	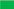	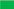	V
71.	ND	−5.7	−0.87	2000	0.55	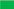	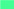	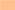	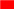	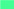	IV
72.	ND	−7.4	0.13	1200	0.17	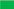	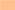	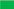	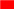	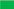	IV

Serial number 69 to 72 represents standard drugs, 69—darunavir, 70—lopinavir, 71—favipiravir and 72—remdesivir; BA, bioavailability score; CG, carcinogenicity; CT, cytotoxicity; LD_50_, fifty percent lethal dose (mg/kg); HT, hepatotoxicity; IT, immunotoxicity; MG, mutagenicity; TC, toxicity class. Different colors mainly represented each chemical’s toxicity profiles; green color represented the chemical is safe/non-toxic, light cyan-color moderates secure, while red color harmful/toxic and soft brick moderate safe for human health.

## Data Availability

The data presented in this study are available on request from the corresponding author.

## Supplementary Materials

The following are available online at https://www.mdpi.com/article/10.3390/biomedicines9111505/s1, Table S1: Physiochemical properties of sixty-eight marine terpenoids with four reference antiviral drugs; Table S2: Predicted pharmacokinetics profiles for marine terpenoids and reference drugs from Swiss−ADME tool.
