# Supplementary material for: Potential of Marine Terpenoids against SARS-CoV-2: An In Silico Drug Development Approach
Source: Biomedicines. 2021 Oct 20;9(11):1505. doi: 10.3390/biomedicines9111505 (PMC8614725; doi:10.3390/biomedicines9111505)
Supplement: Supplementary file 1 [file biomedicines-09-01505-s001.zip › biomedicines-1393784-supplementary.pdf]

Table S1. Physiochemical properties of sixty−eight marine terpenoids with four reference antiviral drugs.

Sl. No.	ChemSpider/ PubChem ID	MW (≤ 500)	XLogP (≤ 5)	H−BD (≤ 5)	HBA (≤ 10)	MR (≤ 130)	RB (≤3)	tPSA (≤ 142)	Synthetic accessibility
1.	15954445	360.5	4.4	0	4	103.86	8	60.4	5.87
2.	—	362.5	3.32	1	4	104.19	3	55.76	6.58
3.	15934381	220.35	2.7	1	1	70.24	1	20.2	4.34
4.	457964	338.5	2.5	4	4	93.70	2	80.9	5.58
5.	186689	442.6	7.2	2	4	132.77	6	66.8	4.87
6.	72185	314.5	6	2	2	97.53	2	40.5	4.08
7.	44139745	436.5	3	1	5	123.90	0	72.8	6.39
8.	6443362	597.1	3.6	1	10	148.88	10	138	7.54
9.	21669867	569	2.8	1	10	139.27	8	138	7.29
10.	21669866	541	2	1	10	129.65	6	138	7.05
11.	21778345	665.3	6.8	2	4	151.12	1	66.8	6.73
12.	46886866	370.5	5.4	1	4	108.41	7	51.8	4.69
13.	27025688	338.44	3.78	1	3	100.74	0	38.69	6.17
14.	122836	218.33	5.3	1	1	71.57	4	20.2	2.47
15.	461032	300.4	3.6	0	2	90.84	1	34.1	4.99
16.	15954444	318.4	4.4	1	3	94.12	6	54.4	5.74
17.	5273523	340.5	5.3	0	3	102.67	9	39.4	4.10
18.	129905343	394.5	4.5	1	5	111.48	5	72.8	5.56
19.	129905666	394.5	4.5	1	5	111.48	5	72.8	5.56
20.	29214894	348.47	4.88	1	4	101.02	3	59	5.24
21.	29214895	364.51	3.66	1	4	104.3	3	55.76	6.35
22.	30771352	378.45	0.95	2	6	99	2	93.06	6.51
23.	13855381	332.4	3.6	2	4	91.48	1	70.7	4.90
24.	156834	412.6	6.7	2	3	123.38	0	49.7	5.16
25.	86577341	535.88	11.93	3	3	169.93	30	69.56	6.09
26.	46848860	288.5	5.7	1	1	93.51	4	20.2	4.87
27.	46848862	288.5	5.5	1	1	95.41	4	20.2	4.72
28.	101695885	354.4	5	0	4	103.70	9	56.5	4.46
29.	8217729	328.45	4.89	0	3	95.22	1	30.99	4.63
30.	8421573	330.46	5.31	0	3	98.37	5	35.53	4.19
31.	11361184	386.6	6	1	3	113.79	4	50.4	5.49
32.	72291	358.5	5.1	1	4	102.64	3	63.6	5.07
33.	44550889	430.5	4.2	1	6	119.84	10	97.7	5.51
34.	10336876	332.4	3.7	2	4	91.48	1	70.7	4.90
35.	20056088	1435.4	−1.20	7	32	300.11	19	502	10.00
36.	20056090	1437.4	−0.93	7	32	300.58	20	502	10.00
37.	6475569	330.4	2.7	1	4	63.6	1	95.19	4.77
38.	10409312	410.6	5.5	2	5	115.80	5	76	6.25
39.	44585437	424.6	6.1	2	5	120.35	5	76	6.36
40.	460367	421.4	6	2	3	110.79	3	57.5	4.21
41.	460368	384.5	4.4	2	4	113.18	5	66.8	4.47
42.	10480473	474.59	4.27	3	7	129.35	5	105.45	5.94
43.	460087	326.4	3.7	0	3	94.19	0	43.4	5.44
44.	11833660	304.5	4.3	0	2	93.69	5	34.1	4.77
45.	13943700	302.5	4.5	0	2	93.22	4	34.1	4.69
46.	101393952	416.5	4.9	3	6	118.56	11	104	4.23

47.	11477080	476.6	5.2	3	7	131.57	8	105	5.86
48.	44575739	476.6	5.2	3	7	131.57	8	105	5.87
49.	129905909	394.5	2.3	1	6	107.55	10	82.1	5.23
50.	6442255	348.4	0.9	1	6	90.13	1	89.9	6.41
51.	3081931	343.5	3.8	2	4	99.46	2	80.4	5.35
52.	21775341	362.5	3.9	1	5	98.98	4	72.8	5.14
53.	42608235	555.1	3.3	2	9	139.14	8	132	7.30
54.	10471525	440.91	1.7	2	7	108.8	2	110	6.24
55.	13855380	332.4	3.6	2	4	91.48	1	70.7	4.90
56.	139583964	338.74	3.7	3	6	85.35	4	96.2	2.53
57.	493326	385.5	3.7	3	4	111.71	0	78.8	5.11
58.	9928112	344.5	6	1	3	102.08	1	38.7	4.95
59.	10984902	605.6	3.6	3	7	153.79	7	97.6	7.32
60.	125904	647.7	4.2	2	8	163.53	9	104	7.24
61.	6481823	472.6	4.8	2	6	135.88	10	93.1	5.01
62.	6481822	472.1	4.8	2	6	135.88	10	93.1	5.01
63.	184606	605.6	3.6	3	7	153.79	7	97.6	7.32
64.	38358410	363.4	5.1	3	3	107.33	1	73.3	3.75
65.	DBS	535.88	11.93	3	3	169.56	30	69.56	6.09
66.	6475568	330.4	2.7	1	4	95.19	1	63.6	4.77
67.	4978425	322.48	3.8	3	3	94	1	61	5.67
68.	6475570	375.5	0.8	1	5	108.17	3	66.8	5.06
69.	213039	547.7	2.9	3	9	149.20	12	149	5.67
70.	92727	628.8	5.9	4	5	187.92	17	120	5.67
71.	492405	157.1	−0.6	2	4	32.91	1	84.6	2.08
72.	121304016	602	1.9	4	13	150.43	14	204	6.33

DBS, designed by the software, ChemDraw Ultra for docking study as no such chemical structure either available in PubChem or ChemSpider databases; HBA, hydrogen bond−acceptors; H−BD, hydrogen bond−donors; MR, molar refractivity (mol/m3); MW, molecular weight (g/mol); RB, rotatable bonds; tPSA, topological polar surface area (Å).

Table S2. Predicted pharmacokinetics profiles for marine terpenoids and reference drugs from Swiss−ADME tool.

**Sl. No.**	**GI−abs.**	**BBB permit**	**P−gp substrate**	**CYP1A2 inhibitor**	**CYP2C19 inhibitor**	**CYP2C9 inhibitor**	**CYP2D6 inhibitor**	**CYP3A4 inhibitor**	**Log K_P (cm/s)**
1.	High	Yes	No	No	No	Yes	Yes	Yes	−5.38
2.	High	Yes	No	No	No	No	No	No	−6.15
3.	High	Yes	No	No	Yes	No	No	No	−5.73
4.	High	No	Yes	No	No	No	No	No	−6.59
5.	Low	No	Yes	No	No	No	No	Yes	−3.90
6.	High	Yes	No	No	Yes	Yes	No	No	−3.97
7.	High	No	Yes	No	No	No	No	Yes	−6.87
8.	Low	No	Yes	No	No	No	No	Yes	−7.36
9.	Low	No	Yes	No	No	No	No	Yes	−7.77
10.	High	No	Yes	No	No	No	No	No	−8.19
11.	Low	No	Yes	No	No	No	No	Yes	−5.51
12.	Low	No	Yes	No	No	No	No	No	−5.51
13.	High	Yes	No	No	No	Yes	Yes	Yes	−4.69
14.	High	Yes	Yes	Yes	Yes	Yes	No	Yes	−5.68
15.	High	Yes	No	No	Yes	Yes	No	No	−6.39
16.	High	Yes	No	No	No	No	Yes	No	−3.89
17.	High	Yes	No	No	Yes	Yes	No	No	−5.55
18.	High	Yes	No	No	No	Yes	Yes	Yes	−5.14

19.	High	Yes	No	No	Yes	Yes	No	Yes	−4.59
20.	High	Yes	No	No	No	No	No	Yes	−5.50
21.	High	Yes	No	No	No	No	No	Yes	−5.50
22.	High	Yes	No	No	Yes	No	No	Yes	−6.33
23.	High	Yes	No	No	No	No	No	Yes	−5.92
24.	High	No	Yes	No	No	No	No	No	−7.93
25.	Low	No	No	No	No	No	No	No	−1.10
26.	High	No	No	Yes	No	No	No	No	−4.08
27.	High	Yes	No	No	Yes	Yes	Yes	No	−4.01
28.	High	No	No	No	Yes	Yes	No	Yes	−4.19
29.	High	Yes	No	No	Yes	Yes	No	Yes	−4.93
30.	High	Yes	No	Yes	No	Yes	Yes	No	−4.83
31.	High	Yes	No	No	No	Yes	Yes	Yes	−4.55
32.	High	Yes	No	No	Yes	Yes	No	Yes	−4.99
33.	High	No	No	No	No	No	Yes	No	−4.42
34.	High	Yes	No	No	Yes	Yes	No	Yes	−4.88
35.	High	No	Yes	No	No	Yes	Yes	Yes	−5.94
36.	High	No	No	No	No	No	No	No	−6.41
37.	High	Yes	Yes	No	No	No	Yes	No	−5.70
38.	Low	No	Yes	No	No	No	No	No	−15.91
39.	Low	No	Yes	No	No	No	No	No	−15.73
40.	High	Yes	No	No	Yes	Yes	No	No	−6.39
41.	High	No	No	No	No	No	No	No	−4.89
42.	High	No	No	No	No	No	No	No	−4.57
43.	High	No	No	No	No	Yes	No	Yes	−4.61
44.	High	Yes	Yes	No	No	Yes	No	Yes	−5.49
45.	High	No	Yes	No	No	No	No	Yes	−6.16
46.	High	Yes	Yes	No	Yes	Yes	No	Yes	−5.69
47.	High	Yes	No	No	Yes	No	Yes	No	−5.11
48.	High	Yes	No	No	Yes	Yes	No	Yes	−4.97
49.	High	No	No	No	No	Yes	No	Yes	−5.34
50.	High	No	Yes	No	No	No	No	Yes	−5.51
51.	High	No	Yes	No	No	No	No	Yes	−5.51
52.	High	No	Yes	No	No	No	Yes	Yes	−7.10
53.	High	No	No	No	No	No	No	No	−7.76
54.	High	No	Yes	No	No	Yes	No	Yes	−5.71
55.	High	Yes	No	No	No	Yes	No	No	−5.72
56.	High	No	Yes	No	No	No	No	Yes	−7.32
57.	High	No	Yes	No	No	No	No	Yes	−7.74
58.	High	Yes	Yes	No	No	No	Yes	No	−5.76
59.	High	No	No	Yes	No	Yes	No	Yes	−5.75
60.	High	Yes	Yes	No	No	No	No	No	−6.00
61.	High	Yes	Yes	No	Yes	Yes	No	No	−4.13
62.	High	No	No	No	No	No	No	No	−7.40
63.	Low	No	No	No	No	No	No	Yes	−7.25
64.	High	No	Yes	No	No	No	Yes	Yes	−5.80
65.	Low	No	No	No	No	No	No	No	−1.10
66.	High	No	No	No	No	No	No	No	−7.40
67.	High	Yes	Yes	Yes	No	Yes	Yes	No	−4.87
68.	High	Yes	No	No	Yes	Yes	No	No	−6.39
69.	Low	No	Yes	No	Yes	Yes	No	Yes	−7.55
70.	High	No	Yes	No	Yes	No	No	Yes	−5.93

71.	High	No	No	No	No	No	No	No	−7.66
72.	Low	No	Yes	No	No	No	No	Yes	−8.62

BBB, blood−brain barrier; GI−abs., gastrointestinal absorption.
